# Forecasting Influenza Epidemics from Multi-Stream Surveillance Data in a Subtropical City of China

**DOI:** 10.1371/journal.pone.0092945

**Published:** 2014-03-27

**Authors:** Pei-Hua Cao, Xin Wang, Shi-Song Fang, Xiao-Wen Cheng, King-Pan Chan, Xi-Ling Wang, Xing Lu, Chun-Li Wu, Xiu-Juan Tang, Ren-Li Zhang, Han-Wu Ma, Jin-Quan Cheng, Chit-Ming Wong, Lin Yang

**Affiliations:** 1 School of Public Health, The University of Hong Kong, Hong Kong Special Administrative Region, China; 2 Shenzhen Center for Disease Control and Prevention, Shenzhen, China; 3 School of Nursing, The Hong Kong Polytechnic University, Hong Kong Special Administrative Region, China; University Hospital San Giovanni Battista di Torino, Italy

## Abstract

**Background:**

Influenza has been associated with heavy burden of mortality and morbidity in subtropical regions. However, timely forecast of influenza epidemic in these regions has been hindered by unclear seasonality of influenza viruses. In this study, we developed a forecasting model by integrating multiple sentinel surveillance data to predict influenza epidemics in a subtropical city Shenzhen, China.

**Methods:**

Dynamic linear models with the predictors of single or multiple surveillance data for influenza-like illness (ILI) were adopted to forecast influenza epidemics from 2006 to 2012 in Shenzhen. Temporal coherence of these surveillance data with laboratory-confirmed influenza cases was evaluated by wavelet analysis and only the coherent data streams were entered into the model. Timeliness, sensitivity and specificity of these models were also evaluated to compare their performance.

**Results:**

Both influenza virology data and ILI consultation rates in Shenzhen demonstrated a significant annual seasonal cycle (*p*<0.05) during the entire study period, with occasional deviations observed in some data streams. The forecasting models that combined multi-stream ILI surveillance data generally outperformed the models with single-stream ILI data, by providing more timely, sensitive and specific alerts.

**Conclusions:**

Forecasting models that combine multiple sentinel surveillance data can be considered to generate timely alerts for influenza epidemics in subtropical regions like Shenzhen.

## Introduction

Influenza is a contagious disease with high transmissibility to spread around the world. Influenza viruses, particularly the type A viruses, are characterized with a high mutation rate. According to WHO, there are about 300,000 deaths related to influenza every year [1]. Although influenza was once considered to be a “cold” disease, influenza-associated disease burden in warm tropical and subtropical regions has been demonstrated as high as in cold temperate regions [2]. For example, in subtropical city Hong Kong, influenza causes more than 1,000 deaths and more than 4,000 hospitalizations of respiratory disease and cardiovascular disease_ENREF_4 every year [3]. Hence it is of great significance for public health to establish a forecasting model with the aim to issue timely warning signals for influenza epidemics. However, unlike temperate countries where influenza virus exhibits one sharp winter peak, tropical and subtropical regions have less clear seasonal pattern and influenza viruses could be active throughout the year [4]. Despite of a few attempts [5], [6], to this date there are no forecasting models that have been widely adopted in tropical and subtropical regions.

With increased awareness on influenza epidemics under warm climates, sentinel surveillance for influenza has been greatly enhanced in subtropical and tropical regions. Shenzhen, one of the largest migratory metropolitan cities located in Southern China, has established the influenza surveillance program in late 1990s [7]. Like many other regions [8], Shenzhen’s surveillance system for influenza includes both clinical surveillance for influenza-like illness (ILI, defined as fever over 37.8°C and/or cough) and laboratory surveillance from a selected sample of ILI patients. Although laboratory surveillance can provide more accurate signals for influenza epidemics, it usually lags weeks, even months, behind clinical surveillance [6]. ILI consultation rates can be quickly and easily collected by clinical doctors to generate more timely alerts, so that health authorities could quickly implement control measures. However, the lack of specific symptoms after influenza infections often resulted in false signals [9]. Previous studies have adopted a variety of forecasting models, such as regression models [10]–[12] and cumulative sum (CUSUM) method [13], [14], to improve the accuracy and timeliness of warning signals, but relatively few attempts have been made in tropical and subtropical regions [6], [15].

Dynamic linear model (DLM) is a type of Gaussian linear state space models used in time series analysis [16], [17]. A previous study using the Hong Kong and US data has found that DLM outperformed regression models and CUSUM method [6]. In this study, we tried to optimize the DLM forecasting model in generating alerts for influenza epidemics in Shenzhen, one of the largest migratory metropolitan cities in mainland China, by integrating ILI surveillance data from different districts.

## Methods

### Data

**Figure 1:**
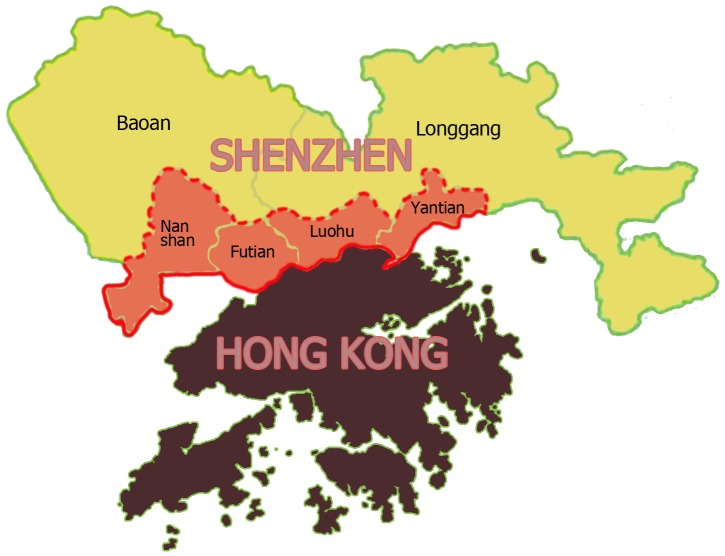
Geographical locations of six districts in Shenzhen. Four districts at the Special Economic Zone are highlighted in red; two suburban districts are highlighted in yellow.

Shenzhen has four districts located at the Special Economic Zone (Luohu, Futian, Nanshan and Yantian) and two suburban districts (Baoan and Longgang) (Figure 1). These districts showed great heterogeneity in terms of living environment, socioeconomic development and population composition (Table S1 in File S1). Weekly proportions of specimens positive for influenza A or B in the entire territory of Shenzhen from 2006 to 2012 were collected by the Shenzhen Center for Disease Control and Prevention (SZCDC) through its sentinel surveillance network established in each district. We defined influenza peak season as the period of at least two consecutive weeks when weekly proportion of specimens positive for influenza exceeded 30% of the maximum level of weekly positive proportions in that year [6], [15]. If there were only one or two nonpeak weeks found between two peaks seasons, they were also classified as peak weeks to form a wide peak. Weekly ILI consultation rate was separately collected in two outpatient settings: 6 general hospitals (GH) and 6 community health centers (CHC), with one in each of six districts [7]. Patients with mild illness often sought medical treatment at community health centers, and relatively severe patients consulted doctors in general hospitals. Two datasets of city-level GH and CHC consultation rates were also calculated by combining the data from all the districts.

### Temporal coherence of surveillance data

Because many respiratory pathogens other than influenza can also cause ILI symptoms, ILI rates are a less specific indicator for influenza virus activity than laboratory data. We decided to assess the coherence between ILI rates and laboratory data in terms of seasonal variation. To deal with non-stationary seasonal patterns shown in most data streams, we used wavelet analysis to assess the temporal coherence of district-level ILI surveillance data and city-level laboratory data. Wavelet analysis has been used to explore the temporal and spatial variations of various infectious diseases including influenza [18], [19]. The advantage of wavelet analysis is that it can decompose time series data into small time-frequency bands to identify the dominant frequencies (cycles) at different time periods. Similar to correlation, wavelet coherence measures the association between two time series at each time-frequency band. High coherence suggests that one time series can be used to predict another. All these 14 data streams of GH and CHC data at district- or city-level showed a similar annual pattern, with the exception of GH data in the Nanshan and Longgang districts. To avoid the false warning signals released from the data streams with distinct seasonal patterns, we excluded the GH data from Nanshan and Longgang from the subsequent analysis. Therefore, a total of 12 data streams were finally included in analysis.

### Dynamic linear model

Dynamic linear model (DLM) [16], [17] is a type of Gaussian linear state space models used in time series analysis. Unlike classical ARMA models, DLMs can be used to model non-stationary time series; therefore it is particularly suitable for the non-stationary surveillance data in our study. We used one of the simplest DLMs, the first order polynomial model, which is also called the random walk plus noise model. This model assumes
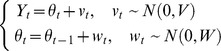



where
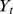
denotes the observed data at week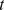, which is a noisy observation for a Markov chain
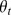
(termed as the state process). Therefore, conditionally on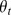, 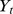
are mutually independent [6], [15], [17]. Based on this assumption,
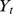
can be predicted from prior conditions and past observations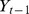. When the observed value of 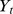
has been obtained, DLM can be updated to make a new forecast value for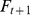. The terms 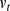
and 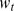
represent observation error and evolution error respectively, under the assumption of mutual independence. Kalman filter can be applied to estimate the variance 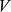
of the data [20], [21]. The parameter 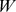
represents the assumed smoothness of the changes of underlying information, i.e., the influenza activity changes from time to time, which are pre-specified as 0.1, 0.075, 0.05, and 0.025 under the assumption of low-to-high smoothness in changes.

### Rules of generating alerts

As the first step, we constructed twelve single-stream models with weekly ILI rates respectively from each district or whole city in either GH or CHC settings. Then multiple-stream models which incorporated all the single data streams of ILI data with similar seasonal patterns were developed.

For single-stream models, an alert was triggered by an aberration, which was defined as weekly observed ILI of any data streams exceeding the upper bound of its forecast interval of 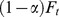 derived from the DLMs. While for the multiple-stream models, a variety of alert rules were adopted and compared: first occurrence of any aberration (R1), 5 simultaneous aberrations (R2), 8 simultaneous aberrations (R3), any 5 aberrations (R4) or any 8 aberrations (R5) first occurred within 2 weeks.

### Model performance

The performance of dynamic linear models using single- and multiple-stream ILI surveillance data was evaluated by sensitivity, specificity and timeliness [22]. Sensitivity measures whether there is at least one alert during each influenza peak season, as



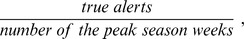



while specificity assesses false alerts generated outside the peak season, as



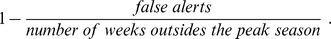
.

Timeliness is defined as the lag time between the first alert and the onset week of each peak season.

To simplify the comparison, we further calculated a single metric of weighted receiver operating characteristic curve (AUWROC) [22], [23], which combined sensitivity, specificity and timeliness together, to measure the overall model performance. AUWROC was calculated as the area under the plot of 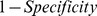 (y-axis) against 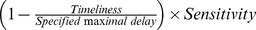 (x-axis), where specificity was set to 95% to simplify calculation. The maximal delay allowed for alerts was 4 weeks [6]. A higher AUWROC value indicated a model with better performance. All analyses were conducted using the R software [24].

We did sensitivity analysis by building dynamic linear models in shorter study periods of 2007–2012 and 2007–2011. To justify our choice of the first-order DLM, we also applied the more complicated second-order DLM to our data [16], [17].

### Ethics

The Ethics Committee of Shenzhen Center for Disease control and Prevention approved this study and written consent was waived by the Ethics Committee of Shenzhen Center for Disease control and Prevention, as there was no personal data involved in this study.

## Results

**Figure 2:**
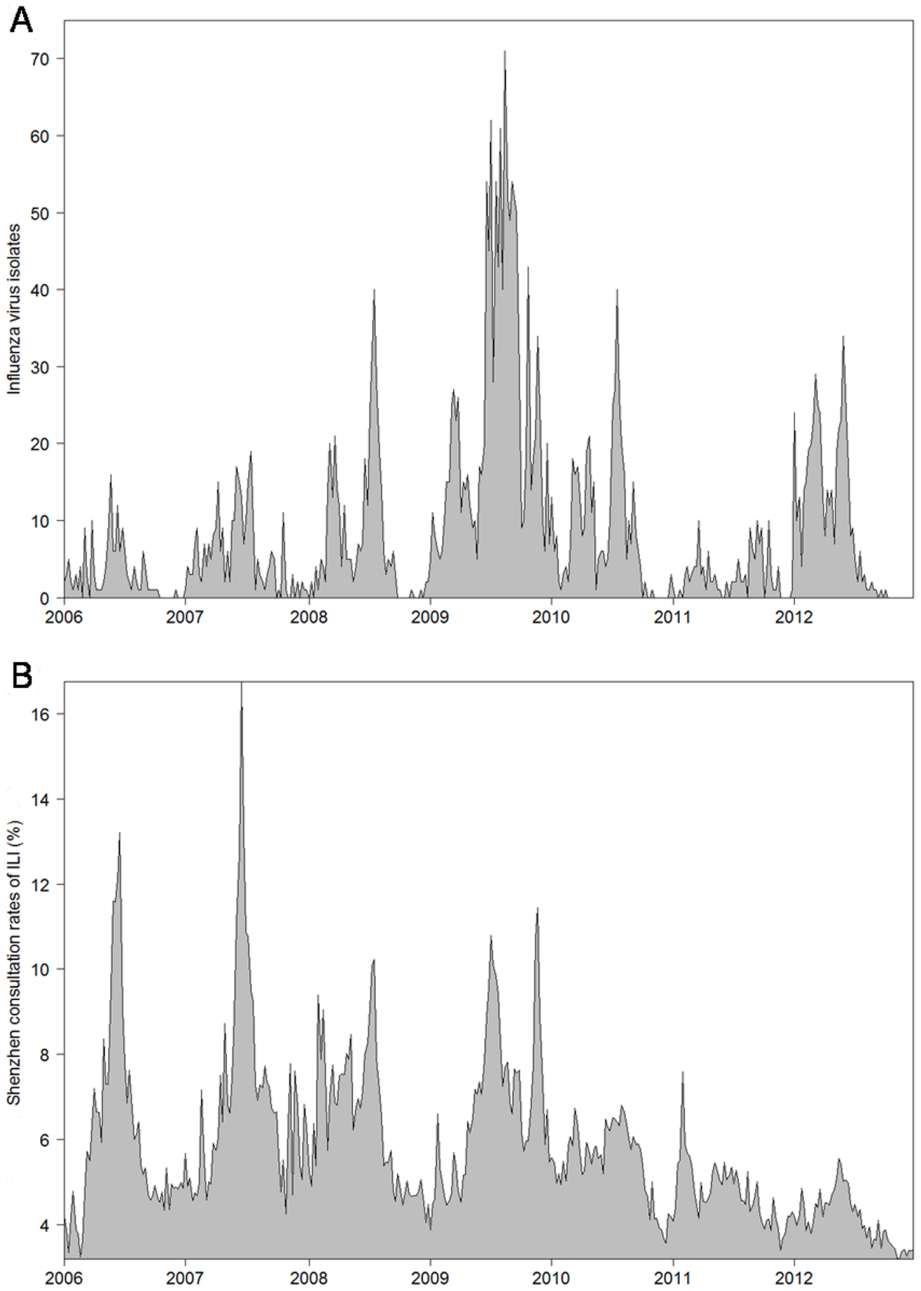
Time series plots of (A) weekly numbers of virus isolates and (B) average ILI consultation rates (%) from both CHC and GH settings, 2006-2012.

**Figure 3:**
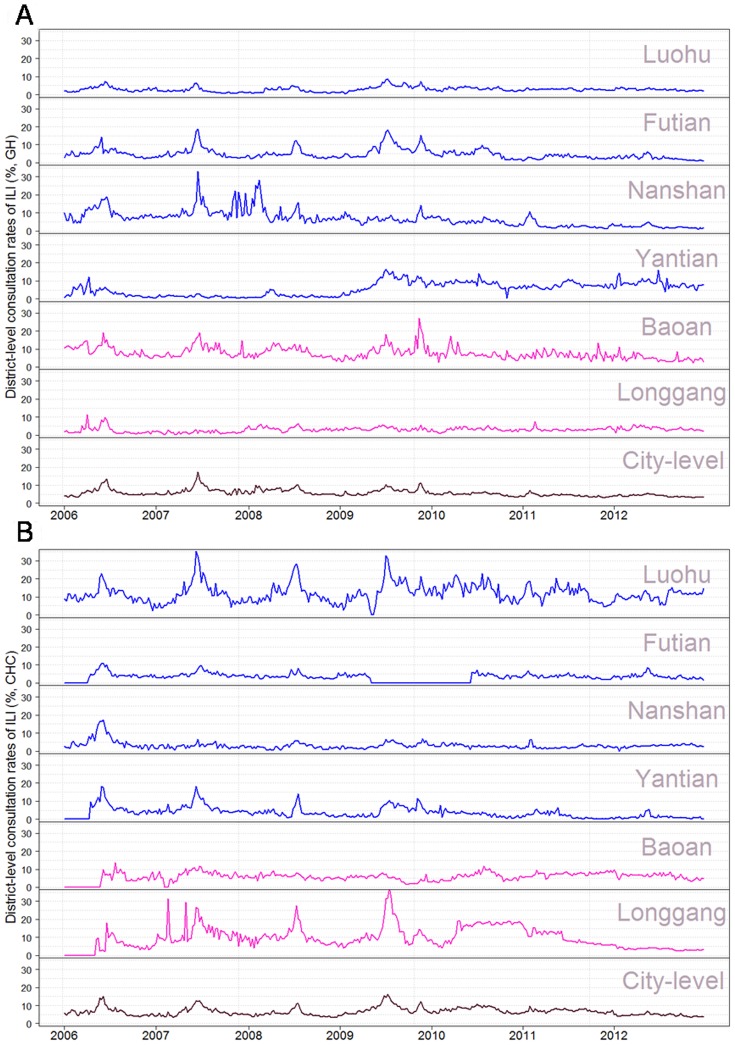
District-level ILI consultation rates, from (A) GH and (B) CHC settings, 2006-2012.

**Figure 4:**
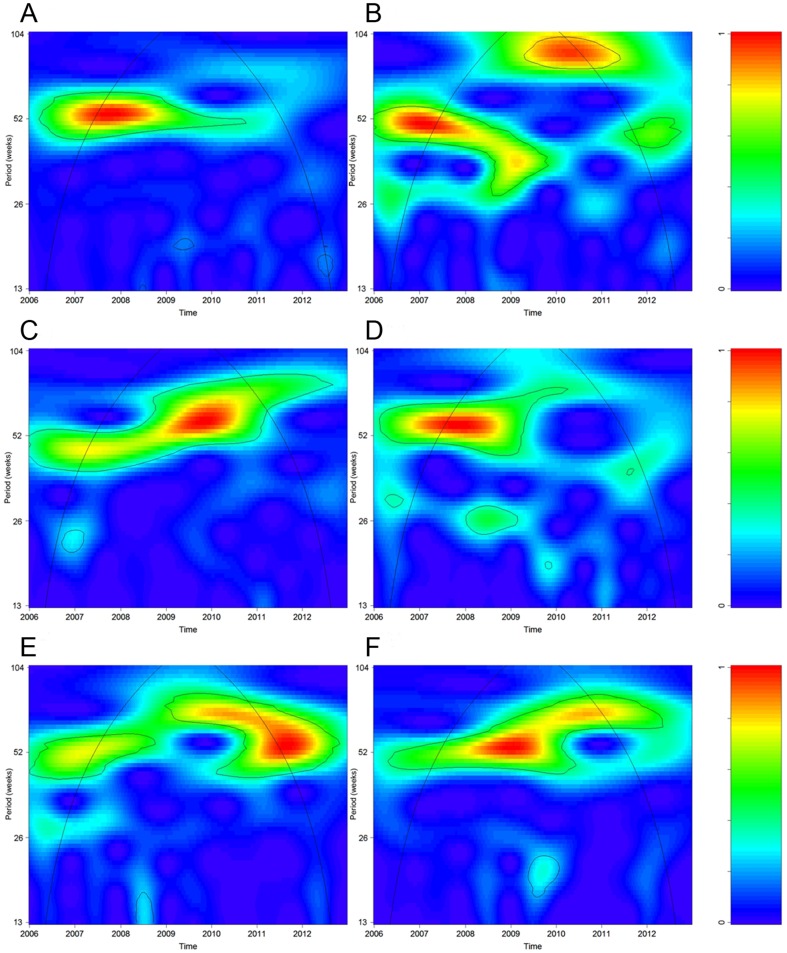
Wavelet spectrums of ILI consultation rates from CHC in six districts, 2006–2012. (A: Luohu; B: Futian; C: Baoan; D: Nanshan; E: Yantian; and F: Longgang). The black contour lines show the regions of time-frequency of the 95% confidence level for the spectrum generated from 1,000 Monte Carlo simulations. The black curve is the cone of influence indicating the region without edge effects. The power values are coded from blue for low power to red for high power in the right panel.

During the study period, we collected 24,210 samples from ILI patients. In total, 3,318 ILI patients were tested positive for influenza by hemagglutination tests. The weekly numbers of influenza positive specimens had an average of 9.1 and range from 0 to 71. Time series plots of city-level laboratory data and average ILI rates are shown in Figure 2, and district-level ILI rates in CHC and GH settings are plotted in Figure 3. Wavelet analysis showed that city-level laboratory data and all district-level CHC-ILI data had a significant annual cycle during the whole study period from 2006 to 2012 (Figure S1 and Figure 4). A similar annual pattern was found for most GH-ILI data, with the only exceptions of Nanshan and Longgang districts where a semiannual cycle occasionally occurred (Figure S2). Significant coherence with laboratory data was found between the remaining 7 single data streams from CHC setting (6 district-level streams plus 1 city-level stream) and 5 from GH setting (4 district-level streams plus 1 city-level stream). These 12 coherent data streams were included for further analysis to ensure that all the data streams follow the similar seasonal pattern.

To facilitate model comparison, we calculated the highest AUWROC, sensitivity and timeliness at a fixed specificity of 95% for each model. For the model with single data stream of each district- or city-level ILI rates, the best performance was achieved by the model with only the GH-ILI data from the Futian district included (AUWROC 0.78 and timeliness 1.18 weeks). The AUWORC of city-level ILI for either GH or CHC settings were 0.74 and 0.71, respectively. In overall, the alerts generated from the single stream models using the GH-ILI data tended to have higher AUWROC than those using the CHC-ILI data (average AUWROC 0.71 vs. 0.65). For the multi-stream models with twelve streams of ILI data, the AUWROC had an average of 0.73 across different alert generating rules. The rule R5, i.e. any 8 aberrations first occurred within 2 weeks, had the most optimal performance, with AUWROC of 0.81 and timeliness of 1.23 weeks. The performance of multi-stream models was better than that of the single-stream models, with higher AUWROC (average 0.73 vs. 0.67), higher sensitivity (average 0.92 vs. 0.83) and less lag time (average 1.54 weeks vs. 2.47 weeks) (Table 1).

**Table 1:** Performance of alerts generated by single monitoring and multiple monitoring by dynamic linear models, Shenzhen, 2006-2012.

	Sensitivity	Timeliness	AUWROC
Single GH#			
Luohu	1.00	2.89	0.72
Futian	0.89	1.18	0.78
Baoan	0.79	5.78	0.72
Yantian	0.64	1.92	0.61
Whole city	1.00	1.31	0.74
Single CHC			
Luohu	0.88	2.25	0.74
Futian	0.69	1.18	0.52
Baoan	0.92	2.47	0.56
Nanshan	0.90	3.14	0.72
Yantian	0.68	3.59	0.62
Longgang	0.53	2.56	0.64
Whole city	1.00	1.41	0.71
Multiple GH+CHC			
R1	1.00	0.08	0.65
R2	0.91	2.16	0.74
R3	0.77	3.50	0.69
R4	1.00	0.71	0.78
R5	0.90	1.23	0.81

Note: GH, CHC.

Sensitivity, timeliness and AUWROC were calculated at a fixed specificity level of 95%;

# General hospitals in Nanshan and Longgang were excluded because their seasonal patterns were different from other districts.

We found that overall the second-order DLM did not obviously improve the model performance, as compared to the simplest first-order DLM (Table S2 in File 1). The first-order DLM that was applied to the data of shorter study periods (2007–2012 or 2007–2011) yielded similar sensitivity, specificity and AUWROC (Table S3 in File S1). In the sensitivity analysis of varying the thresholds of influenza epidemic period, the definition of weekly positive proportion exceeding 40% of annual maximum showed the worst performance, but the definitions of 20% and 30% had similar estimates in all the indicators (Table S4 in File S1).

## Discussion

In this study we compared the performance of different single- or multi-stream DLM using the sentinel surveillance ILI data, in terms of generating sensitive, specific, and timely alerts for influenza epidemics in a subtropical city of Southern China. The better performance of multi-stream models suggests that incorporating different sources of surveillance data could improve the performance of forecasting models. Although there are more complicated multivariate mathematic models available, we used relatively simple DLM in this study, which provides a simple and convenient tool for health authorities in tropical and subtropical regions. It is of note that our model requires that all different data streams follow similar seasonal patterns, which can easily be evaluated by wavelet analysis, as demonstrated in this study.

According to our study, the performance of multi-stream forecasting models with all the twelve ILI data streams for 5 or 8 simultaneous aberrations (R2, R4, and R5) substantially improved in sensitivity, timeliness and AUWROC, as compared with the single-stream model from either GH or CHC settings. We also found that the alert generating rule of 8 aberrations out of total 12 data streams achieved the highest AUWROC. Hence the prediction rule that at least 75% of data streams show aberrations could be considered in future studies on forecasting models to generate an alert for influenza epidemics.

In line with the previous study in Shenzhen, we found that influenza laboratory data and ILI consultation rates in most districts demonstrated annual cycles [25]. However, we conducted wavelet analysis for each district from different surveillance settings of the city, and found the semiannual cycles in Longgang and Nanshan from GH during and after the 2009 pandemic period. The geographical heterogeneity in ILI data might be due to the disparity of socioeconomic development, population composition and health seeking behavior. Unfortunately, there are not many related data available in the district levels. Further studies on the factors that affect geographical heterogeneity are warranted when such data become available.

A potential limitation of our study is the lack of influenza laboratory data in each district, which does not allow us to define district-specific influenza epidemics. Nevertheless, given the highly synchronized seasonal peaks of influenza across even countries [26] and efficient transmission of influenza virus inside the community [27], [28], we believe it is reasonable to assume that all these districts simultaneously entered seasonal influenza epidemics. Another limitation is that we defined influenza epidemics solely based on an artificially set threshold, although sensitivity analysis of using different thresholds suggested that our results were robust to different definitions of influenza epidemics.

In conclusion, we found that forecasting models with multiple data streams of ILI consultation rates could provide more timely and accurate warning signals to influenza epidemics. The modeling strategies of DLM could be applied to other subtropical and tropical regions.

## Supporting Information

Figure S1 **Wavelet spectrums of city-level laboratory data in Shenzhen, 2006-2012.** The black contour lines show the regions of time-frequency of the 95% confidence level for the spectrum generated from 1,000 Monte Carlo simulations. The black curve is the cone of influence indicating the region without edge effects. The power values are coded from blue for low power to red for high power in the right panel.
(TIF)

Figure S2 **Wavelet spectrums of ILI consultation rates from GH in six districts, 2006-2012**. (A: Luohu; B: Futian; C: Baoan; D: Nanshan; E: Yantian; and F: Longgang). The black contour lines show the regions of time-frequency of the 95% confidence level for the spectrum generated from 1,000 Monte Carlo simulations. The black curve is the cone of influence indicating the region without edge effects. The power values are coded from blue for low power to red for high power in the right panel.
(TIF)

File S1 **Supporting Tables.** Table S1. Population and economic indicators of each district in Shenzhen. Table S2. Performance of alerts generated by single monitoring and multiple monitoring using first-order and second-order dynamic linear models during 2006 to 2012. Table S3. Performance of alerts generated by single monitoring and multiple monitoring by dynamic linear models using different study periods. Table S4. Performance of alerts generated by single monitoring and multiple monitoring by dynamic linear models at different thresholds of influenza epidemic period definitions (20%, 30%, or 40%), Shenzhen, 2006-2012.
(DOC)
