# Supplementary figures and images for: Forecasting Influenza Epidemics from Multi-Stream Surveillance Data in a Subtropical City of China
Source: PLoS One. 2014 Mar 27;9(3):e92945. doi: 10.1371/journal.pone.0092945 (PMC3968046; doi:10.1371/journal.pone.0092945)

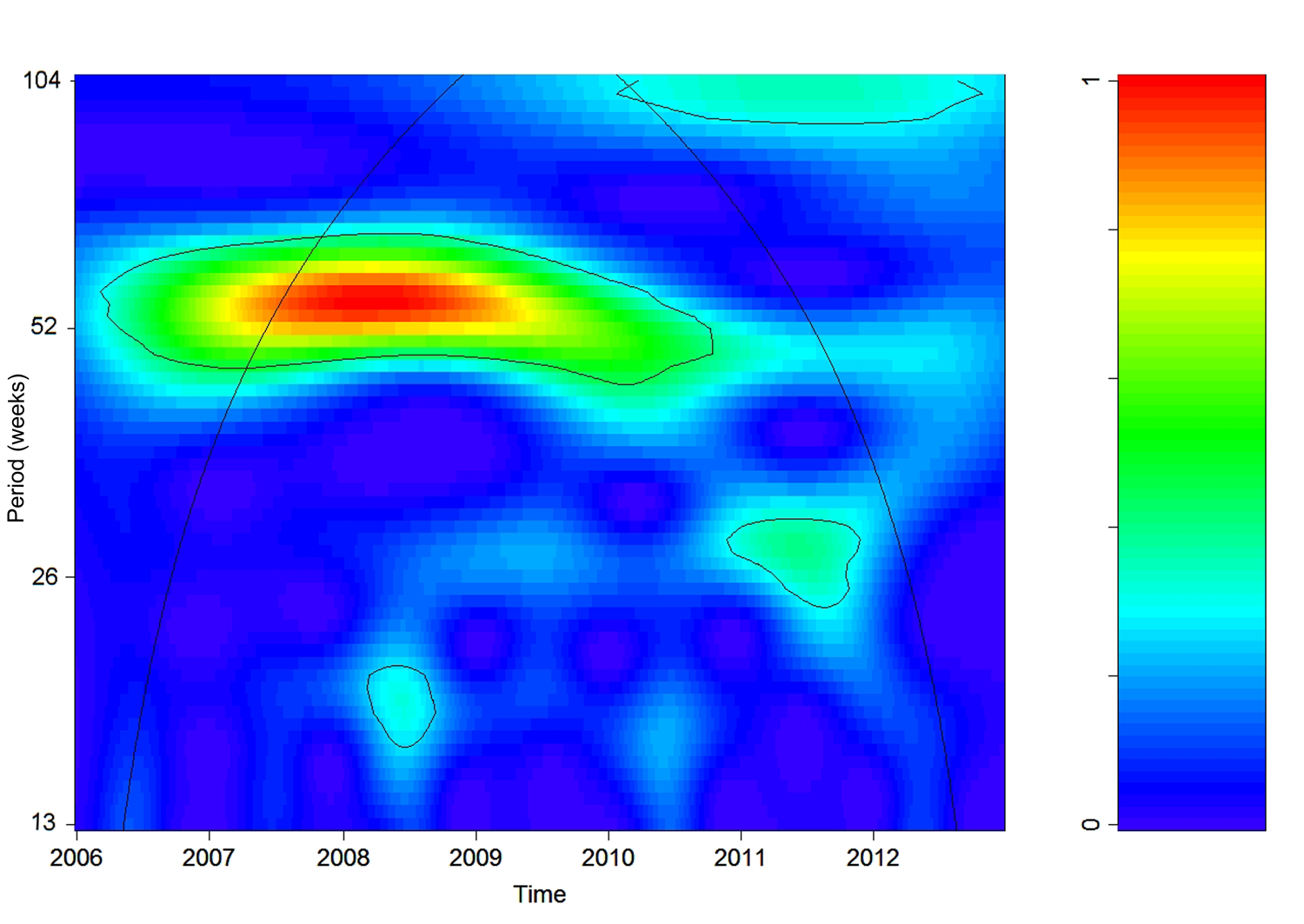

Supplement: Figure S1 — Wavelet spectrums of city-level laboratory data in Shenzhen, 2006-2012. The black contour lines show the regions of time-frequency of the 95% confidence level for the spectrum generated from 1,000 Monte Carlo simulations. The black curve is the cone of influence indicating the region without edge effects. The power values are coded from blue for low power to red for high power in the right panel. (TIF) [file pone.0092945.s001.tif]

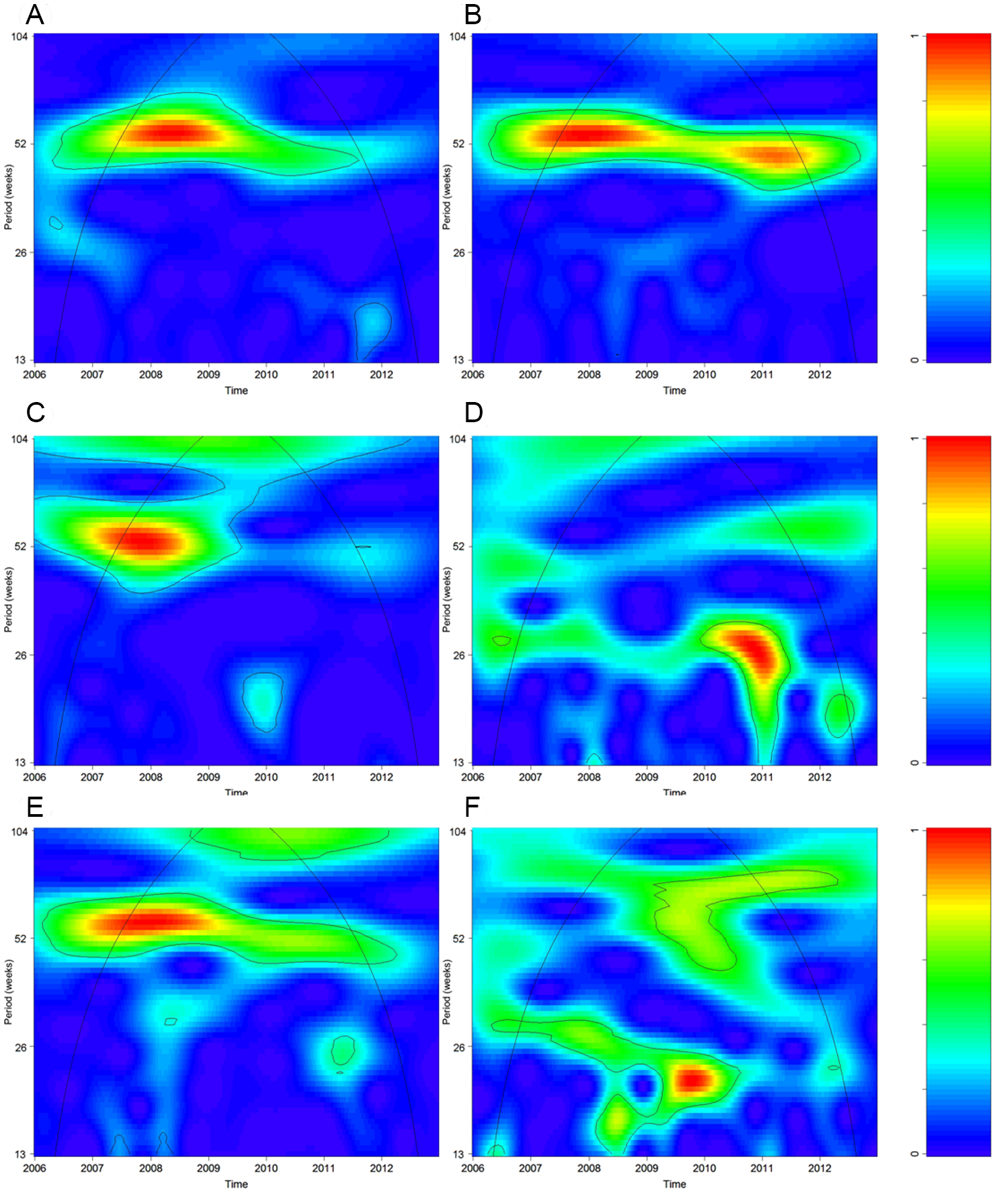

Supplement: Figure S2 — Wavelet spectrums of ILI consultation rates from GH in six districts, 2006-2012. (A: Luohu; B: Futian; C: Baoan; D: Nanshan; E: Yantian; and F: Longgang). The black contour lines show the regions of time-frequency of the 95% confidence level for the spectrum generated from 1,000 Monte Carlo simulations. The black curve is the cone of influence indicating the region without edge effects. The power values are coded from blue for low power to red for high power in the right panel. (TIF) [file pone.0092945.s002.tif]
