# Supplementary material for: Forecasting Influenza Epidemics from Multi-Stream Surveillance Data in a Subtropical City of China
Source: PLoS One. 2014 Mar 27;9(3):e92945. doi: 10.1371/journal.pone.0092945 (PMC3968046; doi:10.1371/journal.pone.0092945)
Supplement: File S1 — Supporting Tables. Table S1. Population and economic indicators of each district in Shenzhen. Table S2. Performance of alerts generated by single monitoring and multiple monitoring using first-order and second-order dynamic linear models during 2006 to 2012. Table S3. Performance of alerts generated by single monitoring and multiple monitoring by dynamic linear models using different study periods. Table S4. Performance of alerts generated by single monitoring and multiple monitoring by dynamic linear models at different thresholds of influenza epidemic period definitions (20%, 30%, or 40%), Shenzhen, 2006-2012. (DOC) [file pone.0092945.s003.doc]

**Table S1.Population and economic indicators of each district in Shenzhen.**

| City/  District or Region | Population (10,000) | Population density (per km^2^) | Non-registered population/Registered population | GDP per capita  (1,000 USD)^&^ |
| --- | --- | --- | --- | --- |
| Shenzhen^#^ | 1,037.20 | 5,21 | 3.13 | 14.76 |
| Futian | 131.96 | 16,78 | 1.15 | 22.44 |
| Luohu | 92.45 | 11,74 | 1.04 | 17.76 |
| Nanshan | 108.94 | 5,89 | 1.29 | 29.36 |
| Yantian | 20.91 | 2,80 | 3.64 | 21.54 |
| Baoan | 450.51 | 6,22 | 7.85 | 9.28 |
| Longgang | 232.43 | 2,74 | 4.62 | 12.35 |

#Source: Shenzhen statistical year book, 2011

**Table S2.Performance of alerts generated by single monitoring and multiple monitoring using first-order and second-order dynamic linear models during 2006 to 2012.**

|  | Sensitivity | Timeliness | AUWROC |
| --- | --- | --- | --- |
| Single GH^#^ |  |  |  |
| Luohu | 1.00 | 3.76 | 0.69 |
| Futian | 0.80 | 2.52 | 0.77 |
| Baoan | 0.72 | 5.31 | 0.69 |
| Yantian | 0.57 | 2.70 | 0.61 |
| Whole city | 0.83 | 1.76 | 0.76 |
| Single CHC |  |  |  |
| Luohu | 0.90 | 2.57 | 0.73 |
| Futian | 0.60 | 2.83 | 0.51 |
| Baoan | 0.92 | 2.85 | 0.55 |
| Nanshan | 0.65 | 1.76 | 0.70 |
| Yantian | 0.61 | 4.27 | 0.56 |
| Longgang | 0.52 | 2.87 | 0.67 |
| Whole city | 1.00 | 3.85 | 0.65 |
| Multiple GH+CHC^$^ |  |  |  |
| R1 | 1.00 | 0.74 | 0.70 |
| R2 | 0.92 | 2.11 | 0.77 |
| R3 | 0.78 | 3.19 | 0.70 |
| R4 | 0.90 | 1.20 | 0.79 |
| R5 | 0.91 | 1.90 | 0.81 |

Note: Sensitivity, timeliness and AUWROC were calculated at a fixed specificity level of 95%; #: General hospitals in Nanshan and Longgang were excluded because of the seasonal pattern different from other districts; $: Rules of generating alerts: first occurrence of any aberration (R1), 5 simultaneous aberrations (R2), 8 simultaneous aberrations (R3), any 5 aberrations (R4) or any 8 aberrations (R5) first occurred within 2 weeks.

**Table S3.Performance of alerts generated by single monitoring and multiple monitoring by dynamic linear models using different study periods**

|  | 2007-2012 | | | 2007-2011 | | |
| --- | --- | --- | --- | --- | --- | --- |
|  | Sensitivity | Timeliness | AUWROC | Sensitivity | Timeliness | AUWROC |
| Single GH^#^ |  |  |  |  |  |  |
| Luohu | 1.00 | 2.03 | 0.76 | 1.00 | 2.11 | 0.76 |
| Futian | 1.00 | 1.55 | 0.74 | 1.00 | 1.64 | 0.72 |
| Baoan | 0.73 | 4.82 | 0.70 | 0.72 | 4.78 | 0.66 |
| Yantian | 0.67 | 1.46 | 0.60 | 0.54 | 0.50 | 0.60 |
| Whole city | 1.00 | 1.55 | 0.74 | 1.00 | 1.78 | 0.72 |
| Single CHC |  |  |  |  |  |  |
| Luohu | 0.84 | 1.98 | 0.74 | 0.83 | 1.93 | 0.76 |
| Futian | 0.73 | 1.86 | 0.52 | 0.70 | 2.09 | 0.48 |
| Baoan | 1.00 | 2.34 | 0.68 | 1.00 | 2.58 | 0.65 |
| Nanshan | 0.90 | 2.98 | 0.76 | 0.87 | 2.54 | 0.76 |
| Yantian | 0.71 | 4.04 | 0.67 | 0.66 | 2.44 | 0.71 |
| Longgang | 0.56 | 3.16 | 0.70 | 0.79 | 2.74 | 0.72 |
| Whole city | 1.00 | 0.33 | 0.68 | 1.00 | 0.50 | 0.75 |
| Multiple monitoring^$^ |  |  |  |  |  |  |
| R1 | 1.00 | 0.00 | 0.58 | 1.00 | 0.00 | 0.61 |
| R2 | 0.90 | 1.76 | 0.78 | 0.89 | 1.50 | 0.81 |
| R3 | 0.74 | 2.42 | 0.77 | 0.85 | 2.13 | 0.80 |
| R4 | 1.00 | 0.15 | 0.79 | 1.00 | 0.47 | 0.78 |
| R5 | 0.90 | 1.13 | 0.82 | 0.88 | 0.90 | 0.84 |

Note: Sensitivity, timeliness and AUWROC were calculated at a fixed specificity level of 95%; #: General hospitals in Nanshan and Longgang were excluded because of the seasonal pattern different from other districts; $: Rules of generating alerts: first occurrence of any aberration (R1), 5 simultaneous aberrations (R2), 8 simultaneous aberrations (R3), any 5 aberrations (R4) or any 8 aberrations (R5) first occurred within 2 weeks.

**Table S4.Performance of alerts generated by single monitoring and multiple monitoring by dynamic linear models at different thresholds of influenza epidemic period definitions (20%, 30%, or 40%), Shenzhen, 2006-201**2.

|  | 20% | | | 30% | | | 40% | | |
| --- | --- | --- | --- | --- | --- | --- | --- | --- | --- |
|  | Sensitivity* | Timeliness* | AUWROC* | Sensitivity* | Timeliness* | AUWROC* | Sensitivity* | Timeliness* | AUWROC* |
| Single monitoring |  |  |  |  |  |  |  |  |  |
| GH^#^ |  |  |  |  |  |  |  |  |  |
| Luohu | 0.85 | 3.67 | 0.58 | 1.00 | 2.89 | 0.72 | 0.80 | 1.99 | 0.71 |
| Futian | 0.71 | 1.41 | 0.78 | 0.89 | 1.18 | 0.78 | 0.88 | 1.73 | 0.68 |
| Baoan | 0.72 | 7.58 | 0.65 | 0.79 | 5.78 | 0.72 | 0.80 | 3.95 | 0.61 |
| Yantian | 0.60 | 1.87 | 0.58 | 0.64 | 1.92 | 0.61 | 0.55 | 2.04 | 0.58 |
| Whole city | 0.82 | 1.95 | 0.69 | 1.00 | 1.31 | 0.74 | 1.00 | 1.49 | 0.66 |
| CHC |  |  |  |  |  |  |  |  |  |
| Luohu | 0.79 | 2.28 | 0.72 | 0.88 | 2.25 | 0.74 | 0.87 | 2.31 | 0.66 |
| Futian | 0.61 | 1.10 | 0.55 | 0.69 | 1.18 | 0.52 | 0.69 | 0.98 | 0.53 |
| Baoan | 0.75 | 3.52 | 0.52 | 0.92 | 2.47 | 0.56 | 0.92 | 2.15 | 0.58 |
| Nanshan | 0.81 | 3.74 | 0.71 | 0.90 | 3.14 | 0.72 | 0.81 | 3.13 | 0.66 |
| Yantian | 0.63 | 4.78 | 0.56 | 0.68 | 3.59 | 0.62 | 0.55 | 3.01 | 0.60 |
| Longgang | 0.53 | 4.07 | 0.60 | 0.53 | 2.56 | 0.64 | 0.50 | 2.15 | 0.60 |
| Whole city | 0.89 | 1.55 | 0.67 | 1.00 | 1.41 | 0.71 | 1.00 | 1.42 | 0.62 |
| Multiple monitoring |  |  |  |  |  |  |  |  |  |
| R1 | 1.00 | 0.50 | 0.63 | 1.00 | 0.08 | 0.65 | 1.00 | 0.54 | 0.55 |
| R2 | 0.72 | 4.16 | 0.66 | 0.91 | 2.16 | 0.74 | 0.63 | 1.90 | 0.70 |
| R3 | 0.76 | 1.24 | 0.73 | 0.77 | 3.50 | 0.69 | 0.97 | 1.35 | 0.71 |
| R4 | 0.77 | 2.88 | 0.68 | 1.00 | 0.71 | 0.78 | 0.91 | 1.87 | 0.68 |
| R5 | 0.81 | 0.84 | 0.74 | 0.90 | 1.23 | 0.81 | 1.00 | 1.23 | 0.66 |

*: The numbers were calculated by fixing specificity to 95%; #: Nanshan and Longgang are excluded because of the different semiannual seasonal pattern
